# The contested politics of de-privatisation and the shifting terrain of the local state: the case of the Ilm-Kreis, Thuringia, Germany

**DOI:** 10.1080/03003930.2023.2298292

**Published:** 2024-01-07

**Authors:** Franziska Christina Paul

**Affiliations:** Adam Smith Business School, University of Glasgow, Glasgow, UK

**Keywords:** Public ownership, de-privatisation, local state, (re)municipalisation, embeddedness

## Abstract

The trend towards de-privatisation has reshaped the role of local governments and their delivery of public services across the world. Local de-privatisation encompasses the twin processes of remunicipalisation, whereby towns, cities, and rural districts take previously privatised services and infrastructure back into public ownership, and municipalisation, a process of setting up new public provision. While global in scope, de-privatisation is particularly pronounced in Germany, prompting debates about the progressive potential of public ownership as an alternative (urban) politics beyond neoliberalism. This paper explores de-privatisation in rural Germany, and critically investigates how the shifting terrain of the local state in the Ilm-Kreis has led to the de-privatisation of two key sectors: waste and bus transportation (and vice versa). The paper illustrates how the two cases unfolded, highlighting the variegated actors and agencies, the complex contexts, and the dynamic and contested politics of de-privatisation in the Ilm-Kreis.

## Introduction

For the past two decades there has been growing interest in processes of de-privatisation and their significance for a pushback against neoliberal governance at the local, municipal, and regional level. On the left, de-privatisation has become a cornerstone of a new pro-public politics aiming to democratise economies and create more socially just, equitable, and environmentally sustainable public services and infrastructure (Chavez and Steinfort 2022; Kishimoto 2020). As such, de-privatisation is studied for its potential to shift ‘common-sense’ understandings in economic and governance thinking, as an ‘actually existing’ rebuke of neoliberal governance in public service delivery, and to explore alternatives to New Public Management and other market-driven logics (Newman 2000). Demands for public ownership have become more mainstream as various crises, from the ongoing and intensifying climate emergency to the COVID-19 pandemic, have exposed the fissures and shortcomings of market-driven approaches and have stressed the need for robust and affordable public services and infrastructure underpinned by democratic public ownership and governance (UCLG 2022). At the same time, processes of de-privatisation are not understood as an end in themselves by those interested in more democratic and accountable forms of local government, and processes of privatisation as well as logics of marketisation and financialisation continue to influence municipal governance models globally (Cumbers and Paul 2022; McDonald and Swyngedouw 2019).

Evident since the early 2000s, the global trend towards de-privatisation has reshaped the role of local governments and the delivery of local public services across the world (Cumbers and Paul 2022). The term de-privatisation is used here as a collective term for various processes which describe the reclaiming of public and essential infrastructure, assets and services delivery from the private sphere, including remunicipalisation and municipalisation, as well as renationalisation (Kishimoto and Petitjean 2017). Remunicipalisation refers to a process whereby towns, cities, and sub-national regions take previously privatised services and infrastructure back into public ownership, while municipalisation is a process of setting up public services and infrastructure where they previously did not exist. This has led to the emergence of regionally and municipally owned state enterprises across a wide range of sectors including water, energy, waste services, transport, education, health and social care, and telecommunications (Hall, Lobina, and Terhorst 2013; Kishimoto 2020; Kishimoto and Petitjean 2017; Paul and Cumbers 2023; Weghmann 2021). Remunicipalisation, municipalisation, as well as renationalisation (which mirrors the process of remunicipalisation at the national scale) are thus part of a wider global trend to *deprivatise* public services and infrastructure (Cumbers 2012, 2021; Cumbers and Paul 2022; Kishimoto 2020; see also: Public Futures 2023,^1^).

While global in scope, the phenomenon of remunicipalisation is particularly pronounced in Germany and has been studied there for more than a decade (Becker, Beveridge, and Naumann 2015; Cumbers and Becker 2018; Engartner 2009; Halmer and Hauenschild 2014; Höffler et al. 2013; Paul and Cumbers 2023; Wagner and Berlo 2017). Utilising material and insights from ongoing empirical work on German remunicipalisation, the paper argues that the complex, dynamic, and contested processes of de-privatisation merit critical and contextual analysis in order to explore the potential of (re)municipalisation as an alternative politics of local governance beyond neoliberalism. In doing so, the paper makes two key interventions in wider remunicipalisation literatures: firstly, applying the lens of the ‘local state’ (beyond the local government alone) to explore the wider contextual conditions and contested dynamics of ownership changes (see also: Duncan and Goodwin 1982), and secondly, disentangling the focus of study, in this case the varied actors and agencies, context, motivations and potential of local de-privatisations. Empirically, too, the paper contributes a novel angle to de-privatisation literatures by exploring rural (re)municipalisation and public ownership processes where most literature to date focusses on urban transitions. The paper presents a case study of the Ilm-Kreis, a rural administrative district in Thuringia, Germany, with a particular history of drastic ownership changes in public provision. Thuringia was part of the German Democratic Republic and thus saw a wave of privatisations of formerly state-owned enterprises and infrastructure following reunification in the early to mid-1990s (Schäfer 2020). Over the past decade, the Ilm-Kreis has seen two major de-privatisation projects, first, the remunicipalisation of waste services in 2015, followed by the municipalisation of public bus transportation services in 2019. As will be discussed in more depth below, the contested but ultimately largely positive experience from the remunicipalisation of waste services had a direct impact on the subsequent municipalisation of bus services in the rural district, which was, however, more severely contested.

While there is now a growing body of research on (re)municipalisation, there is also continuing disagreement about how to assess and evaluate the phenomenon. As Lobina and Weghmann (2021) note, this theoretical and methodological disagreement stems from the fact that different traditions are motivated by different research agendas. The second contribution of this paper builds on this, and further argues that there is still a need to disentangle what, and who, is being studied, as remunicipalisation research often conflates context, motivations, outcomes and potential. With the aim to contribute to the lively debate on de-privatisation presented in this special issue and beyond, the paper thus offers a case study analysis which explores, in rich detail, the unfolding of two processes of de-privatisation in the Ilm-Kreis, Germany, and by drawing out the varied actors and agencies as well as contested dynamics at play across these processes in their particular spatial and socio-political context.

## The de-privatisation debate: theoretical and methodological considerations

The growing literature on de-privatisation has seen lively debate on the underlying context, motivations, and wider potential of processes of remunicipalisation, municipalisation, and, to a lesser degree, renationalisation. In a recent commentary, Lobina and Weghmann (2021) contrast two broader traditions of work on remunicipalisation, positioning the different bodies of work on the issue(s) within heterodox and neoclassical economic thought respectively. Similarly, Cumbers and Paul (2022) identify three perspectives towards studying the nature and potential of de-privatisation across the emerging field of research on remunicipalisation, which can be broadly summarised as a pragmatist apolitical perspective, a critical but sceptical perspective, and a critical progressive perspective. For the purposes of this paper, I will split these into two, contrasting the key arguments and methodological bases of the pragmatist (apolitical) perspective and the critical (progressive) perspective, before offering an intervention that I believe addresses some misconceptions, while grounding the particular approach taken in this paper.

In brief, the so-called pragmatist perspective recognises a rise in in-sourcing but firmly places the phenomenon of remunicipalisation (and other de-privatisation processes) within a periodic pendulum swing between public and private ownership and governance (e.g., Warner and Aldag 2021). In line with technical and economistic theory, this perspective understands de-privatisation as incremental policy change, motivated by managerialist logics, rational economic assessment, and largely void of political influences (Clifton et al. 2019; Warner 2023; Warner and Aldag 2021). On the other hand, the critical perspective opposes the characterisation of (re)municipalisation as an apolitical process, highlighting the inherent complexity of de-privatisation processes and their particular embeddedness in wider social, economic, political, and cultural contexts and relations through drawing on heterodox economic thinking as well as sociological and human geographical theories (Cumbers and Paul 2022). The critical perspective is sometimes conflated with an activist perspective, but while sympathetic to the aims of pro-public movements and alliances, this approach goes beyond merely collecting best practice examples, and critically engages with the wider landscape of public ownership and its (potential) significance for political and economic transformation. Methodologically, too, there has been some disagreement within de-privatisation research on how best to capture the phenomenon of (re)municipalisation. Unsurprisingly, the two perspectives described above propose and utilise different methodological approaches and levels of analyses to arrive at their respective conclusions (Cumbers and Paul 2022; Lobina and Weghmann 2021). The pragmatist perspective relies on quantitative, often large-scale survey data, primarily in a US context (Warner 2023; Warner and Aldag 2021), but with some evidence from Europe (e.g., Clifton et al. 2019). The critical progressive perspective favours mixed methods and qualitative approaches, and argues for the need to contextualise de-privatisation within broader and ongoing multi-scalar processes of political-economic governance (Cumbers and Paul 2022). Analysis takes the form of surveys (see e.g. Public Futures 2023) alongside more in-depth, qualitative case studies (e.g., Becker, Beveridge, and Naumann 2015; Kishimoto 2020; Paul and Cumbers 2023).

I see little point to further entrench and perpetuate a narrative of ‘pragmatist’ versus ‘critical’ remunicipalisation, mostly because, as Lobina and Weghmann (2021) have already pointed out, the two ‘camps’ have differing research agendas. As this is a special issue contribution on remunicipalisation, I offer the following intervention to address some misconceptions and one specific shortcoming of the pragmatist approach, which I criticise here mainly for its narrow view of the conditions and actors involved in de-privatisation (i.e., its overly narrow focus on local government rather than the local state, and the diversity of actors and agencies this involves). Beyond this specific critique, a key misconception in the pragmatist/critical debate is the (un)common point of departure, which, in this paper, is not ‘what is remunicipalisation?’, to which a response might be ‘pragmatic or critical’, but ‘what does remunicipalisation *mean*?’. Few would disagree that local government actors tend to act pragmatically, but this paper argues that they are not the only (nor possibly even the key) actors in remunicipalisation processes, and that looking beyond might reveal widening coalitions of interest for the potential of local public ownership. Without wanting to labour the point, it is rather unsurprising that managers make pragmatic decisions. In fact, if we understand pragmatism as dealing with problems in a practical – as opposed to theoretical or abstract – way, then pragmatism is a key feature of any manager’s job, local government or otherwise. Conducting large scale surveys (as valuable as these are in capturing other data on remunicipalisation) with people who are pragmatists in their professional capacity will likely get ‘pragmatist results’. This, then, is why I critique, and question, firstly, the usefulness of large-scale survey instruments in capturing what are necessarily contextual and relational conditions and processes, and secondly, the value of exclusively focusing on one type of actor (i.e., local government managers; Warner 2023; Warner and Aldag 2021). Pragmatism can, and does, play a role at a decision-making level, but critical approaches would maintain that this does not foreclose politicisation (cf Clifton et al. 2019), nor does it prevent shifting the terrain of the local state in a way that merits further political economic enquiry.

Here, it should be emphasised that most contributors agree that de-privatisation is not an inherently progressive phenomenon. A critical approach aims to highlight the *potential*, not necessity, of (re)municipalisation and de-privatisation to lead to more progressive outcomes, through exploring wider trends and the respective contexts and conditions that de-privatisation occurs in. To emphasise these variegated contextual conditions, this paper utilises the lens of the local state to draw attention to the relational processes and dynamics that impact ownership changes beyond a focus on ‘local government’ (cf Bel, Hebdon, and Warner 2018; Hefetz, Warner, and Vigoda-Gadot 2012). One key intervention of this paper is to stress that while the outcomes of de-privatisation should not be assumed to be more democratic, participatory, and transformative (or alternatively, less market-based and financially oriented), it would be erroneous to assume that this in turn means that *processes* of de-privatisation are value-free and void of political factors and influences. A focus on process, starting with the actors and their varied agencies, the wider context and motivations, and looking at the decision-making, implementation, and further developments, which often tend to be messy and non-linear, allows for a more dynamic understanding of how ownership changes are unfolding in particular places and at particular times than large-scale surveys and quantitative methodologies could allow for (cf. Warner and Aldag 2021). A focus on processes also allows for an appreciation of wider social, political, economic, and cultural factors, as well as the interplay of agency and institutions, and their embeddedness in particular spatial contexts Polanyi, (1957).

This attention to embeddedness is usefully supported by drawing on the concept of the local state (beyond local government). While the hegemony of neoliberal models limited the scope for the local state to pursue alternative political strategies in the last decades of the 20^th^ Century (Geddes 2006; Jessop 2005), critical research into de-privatisation attempts to explore how the return to public ownership is both shaped by, and in turn, shapes the local state in the early 21^st^ century (Paul and Cumbers 2023). The study of public (and collective) ownership, as opposed to previous forms of national, state ownership, also allows for a less curtailed understanding of the kinds of actors and institutions involved in shaping local political economies. Studying processes of de-privatisation through the lens of the local state allows for a more varied ‘cast’ and dynamic interplay between these actors and institutions, moving beyond the pragmatist decision making of ‘local government managers’ (Warner 2023, 2), to include a wide range of stakeholders, from local citizens organising campaigns and referenda, to local politicians on both sides debating issues in council meetings and in public fora, local and multinational businesses variously positioning themselves on issues, and the local, regional, and specialist press and media shaping public discourse.

## De-privatisation in the Ilm-kreis

This paper examines two processes of de-privatisation in the Ilm-Kreis, first looking at waste services, followed by the district’s public bus transportation. The findings detailed below are part of ongoing field research into German (re)municipalisations for the ‘Global Remunicipalisation and the Post-Neoliberal Turn’ (GLOBALMUN) research project, which is funded by the European Research Council (2019-2024). The overarching aim of the GLOBALMUN project is to critically interrogate remunicipalisation and its implications for an emergent post-neoliberal urbanism. The project’s research objectives are, firstly, to develop a conceptualisation of remunicipalisation that captures its diverse spatial, political and social forms; secondly, to assess whether remunicipalisation leads to more progressive forms of state and public action; and thirdly, to critically evaluate the democratic potential of the new forms of municipal public ownership. The paper utilises a case study methodology, including qualitative interviews, event reports and news articles, and grey materials. The interview material utilised in this paper was collected during a field visit to Thuringia, Germany, in January 2020 just prior to the COVID-19 lockdowns. Field research in Thuringia encompassed in-depth semi-structured interviews with 18 experts and stakeholders, event observation, and grey literature analysis. The findings presented in this paper are informed by this wider context, but the paper specifically focuses on the processes and dynamics in the Ilm-Kreis. The paper directly draws on five of the interviews conducted with key stakeholders in the Ilm-Kreis, as well as additional, unstructured interviews with a local government and remunicipalisation expert to supplement interview findings. Additionally, the analysis draws on findings from desk-based research prior to as well as following the visit in Thuringia, including a review of local newspaper articles, specialised press publications, and political and policy documents on the two de-privatisation processes, which was used to supplement (and triangulate) interview findings.

Located just south of the state capital Erfurt, Ilm-Kreis is the fourth largest of Thuringia’s 17 districts (*Landkreise*) and six ‘district-free’ cities (*kreisfreie Städte*) in terms of population with approximately 106,000 inhabitants. German districts are the intermediate level, equivalent to UK or US counties, sitting between state (in this case, Thuringia), and municipal administrations. The Ilm-Kreis unites 16 smaller towns, municipalities, and municipal associations. A district is governed by a district council (*Kreistag*) and the highest-ranking administrative position is that of *Landrat* (male) or *Landrätin* (female). The Ilm-Kreis’s proximity to Erfurt and its central location within both Germany and the EU, alongside well-developed road, rail, and air transport links create favourable conditions for industry, including logistics enterprises, which have given the Ilm-Kreis a comparatively strong economic standing within what is one of Germany’s more deprived federal states.

### Remunicipalising waste services in the Ilm-Kreis

The remunicipalisation of waste management services was originally prompted by a change in European Union legislation aiming to combat cronyism in service awards, which mandated that all fully private or part-private provision of public services, such as waste, needed to be tendered across the EU (Schigold et al. 2017), or alternatively be awarded in-house through a fully public enterprise. In 2010 political and administrative actors in the Ilm-Kreis became aware that the (then) existing contract between the district and its waste company, *Ilmenauer Umweltdienst GmbH* (IUWD, Ilmenau Environmental Services), did not meet legal requirements and required renegotiation. The reason for this was an existing public private partnership between IUWD and the private waste service multinational Remondis, which had not been tendered across the EU as mandated by the law. Remondis, as a private provider, held 49% of IUWD, with the other 51% held by the Ilm-Kreis district (Scheler-Stöhr 2017; Weghmann 2017).

The following account explores the process of deprivatisation of waste services in the Ilm-Kreis, including vacillating decision-making at local government level and a citizen’s referendum, predating the eventual remunicipalisation in 2015. The district began conversations with the relevant regulatory authorities to assess options for waste management services in 2010. Based on the legal changes it was obvious that the existing contract with Remondis as part of IUWD had to be properly terminated but the Ilm-Kreis council had two options going forward. Firstly, to competitively tender the waste management services for the entire Ilm-Kreis on the European market with the risk that the district’s own company (IUWD) might not receive the award, or, secondly, to buy back Remondis’s 49% shares, effectively remunicipalising waste services and thus being able to award them in-house (Rauprich 2020). Despite being involved in the talks from the beginning and being aware of the soon to be illegal ownership structure, Remondis insisted that they would not sell their shares and refused to productively engage in conversations (Scheler-Stöhr 2017; Technologie Region News 2015).

In 2011, the issue was discussed in more depth in the Ilm-Kreis council where political actors initially favoured the competitive tendering option and ordered a feasibility study to be conducted for this first option. However, political actors from the opposition, notably from the left party (DIE LINKE), intervened and proposed to assess the second option, to remunicipalise waste management services instead of the EU-wide tender, citing the risks of rising services charges and worsening service quality following the marketisation of an essential service as had been experienced elsewhere in Thuringia (Interviews with Sascha Bilay and Matthias Gärtner, members of the parliamentary Left Party in Thuringia. January 2020 in Erfurt; see also: Technologie Region News 2012). The ensuing debates and the absence of a handful of pro-tendering politicians during a council session led to a new, small majority in the district council for remunicipalisation in January 2012 (Technologie Region News 2012; Schäfer and; Rethmann 2020). The administration thus re-drafted the existing working documents to reflect the change in policy towards remunicipalisation and again approached the private shareholder Remondis, who, again, rejected the offer by the Ilm-Kreis district to buy back their shares.

The refusal of the private provider to engage with the district complicated matters but did not foreclose a municipally owned waste company in the Ilm-Kreis. However, without the existing resources of Remondis, the Ilm-Kreis district administration had to re-assess the remunicipalisation costing process, and factor in that a new location had to be found (as the existing location would no longer be accessible), and some aspects of waste services had to be re-thought or acquired by the district, including a vehicle fleet and additional staff. However, Ronny Bössel, plant manager of *Abfallwirtschaftsbetrieb Ilm-Kreis*, AIK, the district’s waste management provider points out:

The problematic around additional staff certainly wasn’t the main issue then, as the private provider would have needed to let go of some of their staff who would have been glad to be employed in a new municipal enterprise. The acquisition of a new vehicle fleet however – which would itself have needed to be tendered across the EU – would have been a bigger issue

(Interview with Ronny Bössel, January 2020 in Arnstadt, Thuringia)

The administration proceeded to draft a feasibility study on the remunicipalisation of waste services, which included the additional costs of finding a new location, new staff, and new vehicles, should the private provider continue to refuse to sell their shares. Surprisingly the study concluded that even under this ‘worst case scenario’ of having to start from scratch, waste disposal charges for citizens across the district would not be negatively affected (Interviews with Ronny Bössel and Frank Kuschel; Kuschel 2013; Technologie Region News 2015). The results of the study were a key factor in the decision of the pro-remunicipalisation political actors in the district council to go ahead with plan to establish a publicly owned, municipal waste enterprise, which was passed as resolution 182/12 in the district council (Ilm-Kreis 2014). Based on the political decision, the district administration continued on the path towards remunicipalisation and began searching for a new site for the municipal waste enterprise.

However, in a further turn of events in 2013 (a year on from the remunicipalisation decision), a second motion on the issue was proposed in the Ilm-Kreis district council. Motion 273/13 argued (again) for EU-wide tendering (Ilm-Kreis 2014). The pro-tender motion passed based on a recent change of majorities in the district council, despite the significant groundwork that had been laid by the administration on the conceptualisation and beginning realisation of the remunicipalisation of waste services, including the existing feasibility study, calculations, and preparation for a new location, staff, and vehicle fleet. Subsequently, Ronny Bössel recalls: ‘so then we *had to* – again! – prepare for the European-wide tendering process for the full range of waste services in the Ilm-Kreis’ (Interview with Ronny Bössel, plant manager of AIK. January 2020 in Arnstadt). At the same time, however, the political discussions in the Ilm-Kreis district council continued. The pro-remunicipalisation actors in the council did not want to give up on their vision of a municipal waste enterprise, and a short time later, actors from both the council and, importantly, wider civil society began organising. Civil society actors were supported by various pro-remunicipalisation parties, including the Left Party, the Green Party, the Social Democratic Party, and smaller local parties and voter associations, as well as the German trade union confederation DGB (*Deutscher Gewerkschaftsbund*). The pro-remunicipalisation coalition demanded that the question of what would happen to waste services in the Ilm-Kreis be put to the public for a vote:

‘There was a considerable and wide public discussion with pro and cons but actually it was clear quite early on that the supporters of a municipal waste company were taking the lead […] so a citizen’s referendum was called under the slogan “waste management in public hands”’ (Interview with Ronny Bössel, plant manager of AIK. January 2020 in Arnstadt)

Despite opposition from the bourgeoise-conservative parties (Christian Democratic Union and the Free Democratic Party; see also: Kuschel 2013), including an attempt to delegitimise the referendum, the call for a referendum received the necessary amount of support and was approved by the council and administration (Ilm-Kreis 2014; Scheler-Stöhr 2017). The vote took place on 23 March 2014 with a voter turn-out of 41.47%, high for a rural district council referendum, and a decisive result (more than 70%) in favour of the remunicipalisation of waste services in the Ilm-Kreis (Heß and Buhlemann 2014; Rauprich 2020).

In the context of the clear referendum result for remunicipalisation the council and administration once again focussed on taking waste services provision in the Ilm-Kreis back into public hands. The previous plans for EU-wide tendering were shelved, but the council and administration opted for the part-tendering of three ‘specialised’ services. On the basis of the referendum, renewed talks were held with the private shareholder. Ronny Bössel recalls that the referendum results impressed Remondis, who began to participate more earnestly in negotiations as they feared a loss of image if seen to obstruct a democratic decision. Furthermore, the case of waste services in the Ilm-Kreis had, by then, gained wider media attention across Germany and was followed by the specialised trade press (Technologie Region News 2015). Equally, the *Europäischer Wirtschaftsdienst GmbH* (EUWID), a specialised publisher and European-wide economic information service, followed and covered the case closely (e.g., EUWID 2013, 2019). In this wider context Remondis eventually conceded, and, despite opposing the remunicipalisation in principle, acknowledged the will of the citizens in the Ilm-Kreis by agreeing to sell their 49% of shares, which was finalised in January 2015. Since then, waste services have been upgraded and modernised under public ownership while cost of waste services has not increased (Rauprich 2020; Technologie Region News 2015).

A detailed analysis of the process of waste de-privatisation in the Ilm-Kreis reveals the dynamic and contested politics of remunicipalisation and their particular embeddedness in the local state. By disentangling the various twists and turns of administrative and political decision-making, it becomes clear that the remunicipalisation of waste services in the Ilm-Kreis was shaped by the tensions and dynamic relations of various actors within the rural district, and was eventually realised through the commitment and perseverance of a small majority of official political actors in the district council, as well as supported by a larger majority throughout the district in form of popular citizen support and a successful referendum result. As becomes apparent below, the process and its positively perceived outcome also impacted and shaped the subsequent de-privatisation of bus services in the district in two important ways. On the one hand, a pro-public coalition had been established through the struggle for waste remunicipalisation, and local administrative and political actors had gained more direct experience with de-privatisation, which had shifted the terrain of the local state towards being open to other (re)municipalisation processes, or in other words, more direct democratic control over key services and infrastructure. At the same time, however, opponents of public ownership and delivery, who felt like they had ‘lost’ the debate on waste services, were now more keenly aware of this shifting terrain, which becomes apparent in the more organised and intense contestation of the de-privatisation of bus services discussed below.

### Public transport in the Ilm-Kreis: municipalising bus services

As with the case of waste, a change of European Union legislation also prompted the change of ownership structures of public transport in the Ilm-Kreis. The legislation that initiated the restructuring of bus services in the district was Regulation 1370/2007 of the European Parliament and of the Council regarding public passenger transport services by rail and by road (short: Regulation 1370), which was passed on 23 October 2007.^2^ The regulation entered into force in December 2009, with a period of transitional arrangement of up to ten years to give stakeholders across the EU a chance to adjust rail and road transport services accordingly. Regulation 1370 necessitated an adjustment of public transport services in the Ilm-Kreis. Prior to 2009, transport in the Ilm-Kreis had been provided by two bus companies with similar ownership structures, which had effectively ‘split’ the Ilm-Kreis district between themselves. The southern part of the rural district, including the city of Ilmenau, was serviced by *Omnibusverkehr GmbH Ilmenau* (IOV), while the larger, northern part of the Ilm-Kreis, including the city of Arnstadt, was serviced by *Regionalbus Arnstadt GmbH* (RBA). Both companies were owned by private shareholders, who each held a majority stake of 66%. The respective minority stake of 34% in both companies was held by the *Ilm-Kreis Personenverkehrsgesellschaft mbH* (IKPV, Ilm-Kreis passenger transport company), a 100% subsidiary (public) company of the Ilm-Kreis, which had been established to act as a liaison between the two (private) transport service providers in the rural district. However, while IOV had always been in private hands, RBA had originally been a municipal company, which was privatised in the late 1990s in the context of widespread privatisation of formerly East German public and public-private businesses after German reunification (Rügemer 2006; Schäfer 2020). Based on the guidelines set out within Regulation 1370, the administration of the Ilm-Kreis narrowed down their options for the management of public transport in the district to a choice between competitive tendering or a direct award, also known as an in-house contract, which, however, necessitated the district to fully own, and thus remunicipalise, one or both of the existing transport companies (i.e., IOV and RBA). The choice of options thus mirrors the previous debate concerning waste services in the district.

The following account describes the complex and at times heavily contested process that led to the eventual de-privatisation and restructuring of bus services in Ilm-Kreis in 2019. Following the announcement of Regulation 1370, the Ilm-Kreis council first seriously considered both options (competitive tendering or a direct, in-house award) in 2011. At that time the acting *Landrat* (district administrator), the highest-ranking municipal official in the Ilm-Kreis, was Benno Kaufhold representing the conservative Christian Democratic Union (CDU). Kaufhold and his administration were initially leaning towards competitive tendering. However, *Landrat*-elections in the Ilm-Kreis in May 2012 saw Petra Enders, an independent standing for the left party DIE LINKE, win a run-off vote against Kaufhold with 57.8% of votes.^3^ Following Enders’s election as *Landrätin* in 2012, Ilm-Kreis district council elections in 2014 further shifted the political landscape towards a ‘remunicipalisation-friendly’ council when DIE LINKE became the largest party in the Ilm-Kreis council with a 32.3% share of the vote. Both the shift in political majorities for remunicipalisation (with the Left Party, the social democratic SPD, and the Green Party traditionally in favour of remunicipalisation in the Ilm-Kreis) and the concurrent and positively perceived remunicipalisation of waste management services described above, presented a window of opportunity for the political support of remunicipalisation of bus services (Ilm-Kreis 2020). Crucially, too, the political actors responsible for the decision, i.e., Enders, representatives for DIE LINKE and allies from SPD and the Green Party, were aware of the cautionary tales from Hildburghausen, a city in southern Thuringia, and elsewhere across Germany (see e.g. Mobifair 2020), where the outcomes of EU-wide tendering had led to what interviewees described as ‘Wettbewerbschaos’, so-called ‘competition chaos’, and service quality had suffered as a result (Interviews in Arnstadt, Erfurt, and Ilmenau, January 2020). Following these developments, in early 2015, Lars Sommerfeld, the CEO of IKPV (the fully public subsidiary company of the Ilm-Kreis that liaises with the bus operators in the region) was tasked, by the Ilm-Kreis council, with preparing a concept for the remunicipalisation of bus services to comply with the change of law as required by Regulation 1370. The official discourse around remunicipalisation communicated by the administration and IKPV was deliberately centred on the need to comply with changes to EU legislation. While this could be construed as a ‘purely pragmatic’ shift (Warner 2023; Warner and Aldag 2021), the following account shows how dynamic, and contested, the process of de-privatisation of bus services in the Ilm-Kreis was, and therefore highlights the importance of a contexualised, political economic account of de-privatisation (Cumbers and Paul 2022). On an ideological level, too, the district, under the pro-public leadership of Enders and the Left Party, understood the de-privatisation of bus services and the direct award through a municipal enterprise as an opportunity for a local ‘process of democratisation’ (Interview with Lars Sommerfeld, CEO of IKPV. January 2020 in Arnstadt; see also: Voigtmann 2022).

At first, the de-privatisation of regional bus services seemed straightforward. IKPV was to hold negotiations with the majority owners of both bus operators, RBA in Arnstadt and IOV in Ilmenau. Talks began with the majority shareholder and CEO of RBA, Knut Gräbedünkel, as RBA’s situation was operationally and legally more complex than that of IOV. IOV mainly provided public bus services and only a few other related road transport services (such as ‘one off’ bus trips to drive local pupils to sporting events), while RBA also had ancillary businesses including in timber trade and as a travel agency. RBA’s ancillary businesses presented an obstacle for IKPV, as public monies are not allowed to (cross-)finance private business under German law, and potential public monies for bus services would have risked cross-financing other parts of RBA’s business. As such, negotiations began with RBA to work out how to reconcile the existence of the ancillary businesses with the planned public transport tasks. After one and a half years of negotiations, a contract was put in place. However, while the contract was ready to sign at a notary’s office, RBA failed to show up on the agreed date, leading to a breakdown of negotiations. From mid-2016, IKPV instead focussed exclusively on negotiations with the second bus company, IOV.

The negotiations with IOV were more straightforward and successfully closed in the summer of 2017 when IKPV bought back the full 66% of shares from the private majority stakeholder for just over €900.000. The decision to become a full, 100% shareholder of IOV instead of finding another model (as they had attempted with RBA), was directly influenced by the disappointing and obstructive experience with RBA. The council and administration were also keen to conclude the transition to public ownership as there was growing opposition to changes in relation to Regulation 1370 across the region, and Germany more widely, from the private bus and coach industry, its lobbying actors, as well as the Thuringian charter of the Chamber of Industry and Commerce (Bulut 2019; Voigtmann 2022). In light of these developments, IKPV decided that full ownership of the company shares was the preferable and more risk-adverse option for the district, which ultimately gave the Ilm-Kreis the power to directly award public bus transport to IOV as their own, in-house company. In an interview with Matthias Höring, who had been the majority private owner of IOV and retained the role of CEO following the municipalisation, Höring explained that he realistically assessed his options after IKPV approached him with a remunicipalisation offer:

‘If I would have fought [the offer], the situation in the Ilm-Kreis would have turned out as it did in Hildburghausen and Gotha [other cities in Thuringia]. There would have been EU-wide competitive tendering, because in the short amount of time that the district had, they couldn’t have created their own municipal company from scratch. And, well, then we would have had to face the European competition and would have suffered the consequences with big players such as Veolia and Deutsche Bahn. […] So, to avoid that uncertainty and the stress for myself personally, I decided in agreement with my family that I sell my 66% of shares and thereby help lay the groundwork for the Ilm-Kreis to remunicipalise public transport services’. (Interview with Matthias Höring. January 2020 in Ilmenau)

While Höring, as the private owner, was not a driving force behind the process, he understood the implications of both the Regulation 1370 and the political decisions that had been made in the district council. To put it in a narrowly economistic perspective, he was aware of the business implications of the process, especially that a tendering option might put him out of business, or at least into competition with the European market. He was also personally aware of cases across Thuringia, which had faced disruption and uncertainty following an EU-wide tender (referring to the previously discussed case of Hildburghausen). Notwithstanding the fact that he decided in his (and his family’s) economic interest when selling his shares, Höring’s analysis of the situation also shows a concern for the local community and wider region in line with thinking around community and regional wealth and value creation and the specifically German context of *Daseinsvorsorge* (‘public (well)being provision’) addressed elsewhere (see: Paul 2020; Paul and Cumbers 2023). It is of course unsurprising that private shareholders might be ‘reluctant’ supporters of de-privatisation, but it should not be assumed that business owners, especially of small to medium enterprises who are locally embedded (such as transport providers) automatically value individual profit over the needs of their local communities (Wright 2019). Further, while Höring’s continued involvement as CEO might appear, at first, a ‘business as usual’ scenario under New Public Management, it becomes clear below that the council is proactively involved in shaping the strategy whilst retaining Höring’s decade-long expertise, connections, and knowledge about running bus services in the rural district. A key point here is that locally embedded actors, such as Höring, can be enrolled in remunicipalisation campaigns and are often persuaded to do so by their own and their commumities’ local or regional wealth building interests (see also: Cumbers and Paul 2020).

IOV became a publicly owned, municipal transport company on 1 January 2018, and has since been an indirect investment of the Ilm-Kreis, as the district holds 100% of the company shares of IKPV, which in turn holds 100% if the shares of IOV. With IOV’s municipalisation on 1 January 2018, the Ilm-Kreis was technically one and a half years ahead of the deadline set by the ten-year transitional period for Regulation 1370 (which ended on 1 July 2019). However, according to the federal German Passenger Transport Act (*Personenbeförderungsgesetz*),^4^ prior to a direct award of transport services, the market needs to be ‘informed’ through an obligatory preliminary announcement which has a mandatory 12 month announcement period. Between 1 January 2018 and 1 July 2019, the date of the de-privatisation and the service start date, some changes had to be made to IOV as it transitioned from a majority private to a fully public municipal transport enterprise. As IOV had previously covered a much smaller area (only serving the southern part of the Ilm-Kreis district), the company had to recruit additional staff and fleet vehicles. The company grew from 70 employees prior to the municipalisation process to a workforce of 120 at the time of the research (January 2020). When asked how IOV and IKPV recruited employees for the new routes (which largely corresponds with the routes previously covered by RBA) in light of the failed negotiations with RBA, Lars Sommerfeld explained:

‘We obviously attempted to recruit employees from [RBA] as far as possible, but it wasn’t easy – we received two injunction suits [from RBA] for labour piracy. But, well, from the 50 additional employees we hired about 35 are from RBA here in Arnstadt. So we only had to recruit about 15 new members of staff’ (Interview with Lars Sommerfeld, CEO of IKPV. January 2020 in Arnstadt).

Injunction suits for labour piracy were not the only challenges in this period of adjustment, when the contestation of the de-privatisation of bus services reached new heights (Scheler-Stöhr 2017). While the remunicipalisation of waste management services was not without friction and required careful dialogue (as explored above), the remunicipalisation of bus services was heavily fraught with conflict. Following RBA’s no-show at the notary’s office, the process faced intense opposition from RBA, some parts of the local and specialist press (see e.g. Bulut 2019), as well as political actors from the conservative CDU, centre to centre-right Freie Wähler and FDP, and far-right Alternative for Germany (AfD). RBA’s management was locally embedded in cultural and political structures in Arnstadt and had established connections to parties that opposed the de-privatisation (Interviews with Lars Sommerfeld and Frank Kuschel, January 2020). The conflict between RBA and the rural district council and administration played out in various ways: firstly, in an attempt to stop the direct award of bus transportation services to IOV as a new municipal transport enterprise, RBA had brought a legal challenge before the Higher Regional Court (*Oberlandesgericht*) in Jena (Glinski 2019). Secondly, there was strong anti-remunicipalisation coverage in the local press, which was heavily concentrated on, and vilified, *Landrätin* Petra Enders (Bulut 2019). Finally, and relatedly, there was political opposition from the traditionally bourgeois-conservative and right-wing parties in the council (including CDU, FDP, Freie Wähler, as well as AfD). It should be noted that all conflict was resolved within less than half a year of service provision and the legal challenges were ultimately decided in favour of IKPV and the Ilm-Kreis. As such, the de-privatisation of bus services is seen as a satisfactory, even successful, project across the Ilm-Kreis district (Interviews with Lars Sommerfeld and Frank Kuschel, January 2020).

There might be nothing unexpected in opposition to de-privatisation from a private provider about to lose their business or, for that matter, from bourgeois-conservative media and politicians. However, the unfolding and intense conflict did shape the process in interesting ways. The opposition, threats, and legal challenges made actors in administration and council more resolved to ‘future-proof’ the municipalisation. By choosing a direct award, the Ilm-Kreis council and administration, who had previously communicated a neutral shift in ownership to adhere to EU Regulation 1370, then clearly decided against competitive tendering, free market-orientation, and asset competition for their local and regional public transport provision. Lars Sommerfeld explains:

‘We wanted to have direct control over a topic as complex as public transport – which also becomes increasingly important anyway – especially when we are investing so much of the district’s financial capital into it, we want to directly exert influence, we want to be able to *shape* it. Politically, in the district, public transport is also understood as more than just getting pupils from A to B, it is also always understood as an integral part of our climate protection strategy. And that’s why it was actually quite obvious: “no, we do not want market competition here, because it isn’t clear who actually provides the services in the end and how … and we also do not want wage competition at the expense of the employees!”. So, we wanted to gain the freedom to design public transport our way – we wanted direct influence’ (Interview with Lars Sommerfeld, CEO of IKPV. January 2020 in Arnstadt).

The potential precariousness of free market provision and the associated risks for service delivery and local employment conditions were heightened during the heavily contested process, which led to actors realising the importance of being able to shape services through democratic decision-making and control. Importantly, the quote above does not just speak to the main theme of de-privatisation and public ownership as a means to take back control from the volatility of free market economics, but also highlights important intersections to wider political strategies and policy, specifically speaking to climate and environmental concerns as well as fair labour and employment practices. The council and IKPV identified the need for a public transport enterprise to bring stability for the region through current and future crises. The public ownership and municipal management of transport services, for example, allows councils and administrations to develop comprehensive policy on climate protection and climate change adaptation, in the Ilm-Kreis and beyond (Paul and Cumbers 2023, forthcoming). Interestingly, too, the quote emphasises the value of having both freedom and scope to design policy and strategies differently the Ilm-Kreis, from climate change adaptation to worker’s rights and job security.

## Conclusions

On a superficial level, the two processes of de-privatisation in the Ilm-Kreis could be read as pragmatic decision-making of municipal actors followed by, in the case of bus services, a business-as-usual management of the newly public assets (cf Warner 2023). After all, the context, a change of law at EU level, initiated the decision to remunicipalise waste and bus services, and, in the case of bus services, the former owner retained a position as CEO. In contrast to remunicipalisations in Berlin or Hamburg (Becker, Beveridge, and Naumann 2015; Cumbers and Becker 2018), which saw high levels of civil society and activist involvement and bottom-up, broad-based campaigning from the beginning, the motivations and (initial) outcomes in the Ilm-Kreis *seem to* align more closely with a pragmatist perspective, or what others may even describe as ‘apolitical’ (Clifton et al. 2019; Warner and Aldag 2021). These findings would certainly have shown up in a quantitative survey methodology, and potentially underlined such an assessment. However, an in-depth, qualitative exploration of the two processes exposes the dynamic, contested, and inherently political nature of the de-privatisation of both waste and bus services in the Ilm-Kreis, and sheds light on the shifting terrain of public provision. Studying processes of de-privatisation in the Ilm-Kreis in their rich and contextual detail, including the vacillating decision-making, combinations of proponents and opponents, their agencies and dynamic interplay with more formal institutions, uncovers that de-privatisation in the Ilm-Kreis encompasses more than pragmatist decision-making by official municipal actors, and, in fact, involves a wide range of stakeholders who are shifting the terrain of the local state towards public ownership.

Researching remunicipalisation through the concept and lens of the local state, beyond the local government, helps to disentangle some of the dynamic processes involved. There are, of course, core institutions of the local state, such as the local government, administration, and businesses, which played a key role in the de-privatisations of waste and bus services in the Ilm-Kreis. However, the local state is made up of more actors than the commonly recognised institutions within it (Duncan and Goodwin 1982). Instead of looking only at the response of ‘official’ municipal actors (be that pragmatic, apolitical, critical, progressive, or otherwise) (cf. Warner 2023), there is a need to consider what types of other actors and organisations are involved, and how, in these deeply embedded and spatially variegated processes (see also: Cumbers and Paul 2022). With the case of waste services, there was a key involvement of civil society through a campaign for de-privatisation which culminated in a successful referendum. The campaign was made up of citizens, workers, local parties and politicians, as well as trade unionists, who, together, attempted and succeeded to shift the terrain of local democracy, and achieved the remunicipalisation of waste services. For the case of bus services, there was an even more varied landscape of stakeholders, comprising many of the previous actors and institutions, as well as the involvement of local, regional and specialist press and media, the courts and legal system, and a (locally embedded) private business.

The remunicipalisation of waste services held clear generative potential for the subsequent de-privatisation process of public transport. The context, motivations, and outcomes of the two de-privatisation processes are complex, contested, embedded and dynamic (Polanyi 1957), and the processes themselves shape, and are shaped by the specific terrain of the local state. Focusing on a diverse array of actors and agencies and paying attention to their specific embeddedness, as well as to the variegated and relational nature of the local state (Jessop 2005), highlights widening coalitions of interest around local public ownership, and by extension, around the potential for local economic development through ownership and governance changes. This potential also extends beyond the economic sphere to concerns about environmental sustainability and worker’s rights, as evidenced in the Ilm-Kreis, signalling to a wider alternative politics of local public ownership and governance beyond neoliberalism.

## Notes

1. The Public Futures database presents the largest survey and collection of global cases of de-privatisation to date. The database can be accessed at: https://publicfutures.org

2. Legal text for Regulation 1370/2007 of the European Parliament and of the Council: https://eur-lex.europa.eu/legal-content/EN/TXT/PDF/?uri=CELEX:32007R1370&from=DE

3. Results of the 2012 election of *Landräte* of district-free cities in Thuringia: https://wahlen.thueringen.de/datenbank/wahl1/WAHL.asp?wahlart=LR&wJahr=2012&zeigeErg=WK&auswertung=1&wknr=070&gemnr=&terrKrs=&gemteil=000&buchstabe=&Langname=&wahlvorschlag=&sort=&druck=&XLS=&anzahlH=0&Nicht_existierende=&x_vollbildDatenteil=&optik=&aktual=&ShowLand=&ShowWK=&ShowPart=

4. Legal text (German original) of the German Passenger Transport Act (*Personenbeförderungsgesetz*): https://www.gesetze-im-internet.de/pbefg/BJNR002410961.html

## References

[cit0001] Becker, S., R. Beveridge, and M. Naumann. 2015. “Remunicipalisation in German cities: Contesting neoliberalism and reimagining urban governance?” *Space and Polity* 19 (1): 76–90. 10.1080/13562576.2014.991119.

[cit0002] Bel, G., R. Hebdon, and M. Warner. 2018. “Beyond Privatisation and Cost Savings: Alternatives for Local Government Reform.” *Local Government Studies* 44 (2): 173–182. 10.1080/03003930.2018.1428190.

[cit0003] Bulut, A. 2019. “Landrätin terrorisiert Gräbedünkel/1,5 Millionen fehlinvestiert?” *Busblickpunkt*, April 19. https://www.busnetz.de/buschfunk/landraetin-terrorisiert-graebeduenkel-15-millionen-fehlinvestiert/.

[cit0004] Chavez, D., and L. Steinfort. 2022. “The Future is Public! The Global Reclaiming and Democratization of Public Ownership Beyond the Market.” *Development* 65 (2–4): 207–216. 10.1057/s41301-022-00350-3. 36320617 PMC9610302

[cit0005] Clifton, J., M. Warner, R. Gradus, and G. Bel. 2019. “Remunicipalisation of public services: Trend or hype?” *Journal of Economic Policy Reform* 24 (3): 293–304. 10.1080/17487870.2019.1691344.

[cit0006] Cumbers, A. 2012. *Reclaiming Public Ownership: Making Space for Energy Democracy*. London: Zed.

[cit0007] Cumbers, A. 2021. *The Case for Economic Democracy*. Cambridge, UK: Polity Press.

[cit0008] Cumbers, A., and S. Becker. 2018. “Making Sense of Remunicipalisation: Theoretical Reflections on and Political Possibilities from Germany’s Rekommunalisierung Process.” *Cambridge Journal of Regions, Economy & Society* 11 (3): 503–517. 10.1093/cjres/rsy025.

[cit0009] Cumbers, A., and F. Paul. 2020. “Adapting to the Political Moment and Diverse Terrain of ‘Actually Existing Municipalisms’.” *Soundings* 74 (3): 40–53. 10.3898/soun.74.03.2020.

[cit0010] Cumbers, A., and F. Paul. 2022. “Remunicipalisation, Mutating Neoliberalism and the Conjuncture.” *Antipode* 54 (1): 197–217. 10.1111/anti.12761.

[cit0011] Duncan, S., and M. Goodwin. 1982. “The Local State and Restructuring Social Relations: Theory and Practice.” *International Journal of Urban and Regional Research* 6 (2): 157–308. 10.1111/j.1468-2427.1982.tb00572.x.

[cit0012] Engartner, T. 2009. “Kehrt der Staat zurück? Rekommunalisierungen in den Aufgabenbereichen Entsorgung und Gebäudereinigung.” *Zeitschrift für öffentliche und gemeinwirtschaftliche Unternehmen* 32 (4): 339–355. 10.5771/0344-9777-2009-4-339.

[cit0013] EUWID. 2013. “Ilmkreis: Kommunalisierung oder Ausschreibung?” Issue 47/2013, November 19. https://www.euwid-recycling.de/news/wirtschaft/ilmkreiskommunalisierung-oder-ausschreibung-191113/

[cit0014] EUWID. 2019. “Kommunalisierung auch nach Jahren noch von Interesse”. Issue 12/ March 19, 2019. https://www.euwid-recycling.de/news/politik/kommunalisierung-auch-nach-jahren-noch-von-interesse-190319/

[cit0015] Geddes, M. 2006. “Neoliberalism and Local Governance.” *Policy Studies* 26 (3–4): 359–377. 10.1080/01442870500198429.

[cit0016] Glinski, S. 2019. “Ilm-Kreis: RBA Klage vor OLG Jena.” *Roter Renner*, May 15. https://roter-renner.de/no_cache/detail/datum/2019/05/15/ilm-kreis-rba-klage-vor-olg-jena.html.

[cit0017] Hall, D., E. Lobina, and P. Terhorst. 2013. “Re-Municipalisation in the Early 21st Century: Water in France and Energy in Germany.” *International Review of Applied Economics* 27 (2): 193–214. 10.1080/02692171.2012.754844.

[cit0018] Halmer, S., and B. Hauenschild. 2014. *Remunicipalisation of Public Services in the EU*. Vienna: ÖGPP.

[cit0019] Hefetz, A., M. Warner, and E. Vigoda-Gadot. 2012. “Privatization and Intermunicipal Contracting: The US Local Government Experience 1992-2007.” *Environment & Planning C Politics & Space* 30 (4): 675–692. 10.1068/c11166.

[cit0020] Heß, A., and F. Buhlemann. 2014. “Mehrheit im Ilmkreis stimmt für eine kommunale Müllentsorgung.” *Thüringer Allgemeine*, March 24. https://www.thueringer-allgemeine.de/politik/mehrheit-im-ilmkreis-stimmt-fuer-eine-kommunale-muellentsorgung-id219970031.html.

[cit0021] Höffler, F., C. Schaefer, U. Papenfuß, M. Rosenfeld, and G. Landsberg. 2013. “Rekommunalisierung: Renaissance öffentlicher Unternehmen?” *Wirtschaftsdienst* 93 (2): 71–86. 10.1007/s10273-013-1489-1.

[cit0022] Ilm-Kreis. 2014. “Abfallwirtschat in kommunaler Hand.” Official website: https://www.ilm-kreis.de/Landkreis/Bürgerservice/Bürgerbeteiligung/Abfallwirtschaft-in-kommunale-Hand/.

[cit0023] Ilm-Kreis. 2020. “Ilmenauer Umweltdienst leiset seit 30 Jahren hervorragende Arbeit.” Official website: https://www.ilm-kreis.de/Quicknavigation/Startseite/Ilmenauer-Umweltdienst-leistet-seit-30-Jahren-hervorragende-Arbeit.php?object=tx,2778.5&ModID=7&FID=2778.2476.1.

[cit0024] Jessop, B. 2005. “The Political Economy of Scale and European Governance.” *Tijdschrift voor Economische en Sociale Geografie* 96 (2): 225–230. 10.1111/j.1467-9663.2005.00453.x.

[cit0025] Kishimoto, S. 2020. *The Future is Public: Towards the Democratic Ownership of Public Services*. edited by Olivier Petitjean. Lavinia Steinfort. Amsterdam: Transnational Institute.

[cit0026] Kishimoto, S., and O. Petitjean, eds. 2017. *Reclaiming Public Services: How Cities and Citizens are Turning Back Privatisation*. Amsterdam: Transnational Institute.

[cit0027] Kuschel, F. 2013. “Schlafstörungen wegen der Kommunalisierung der Abfallwirtschafts unbegründet.” Die Linke Kreisverband Ilm-Kreis, 16 January: https://www.die-linke-ilmkreis.de/nc/presse/pressemitteilungen/detail/news/schlafstoerungen-wegen-der-kommunalisierung-der-abfallwirtschaft-unbegruendet.

[cit0028] Lobina, E., and V. Weghmann. 2021. “Commentary: The Perils and Promise of Inter-Paradigmatic Dialogues on Remunicipalisation.” *Journal of Economic Policy Reform* 24 (3): 398–404. 10.1080/17487870.2020.1810473.

[cit0029] McDonald, D., and E. Swyngedouw. 2019. “The New Water Wars: Struggles for Remunicipalisation.” *Water Alternatives* 12 (2): 322–333.

[cit0030] Mobifair. 2020. “Bus-Chaos Landkreis Konstanz – Problemlösung vertagt.”, February 14: https://www.mobifair.eu/2020/02/bus-chaos-landkreis-konstanz-problemloesung-vertagt/.

[cit0031] Newman, J. 2000. “The New Public Management.” *Local Government Studies* 26 (1): 97–100. 10.1080/03003930008433980.

[cit0032] Paul, F. 2020. “Exploring the Role of ‘The Public’ in Social Economics: Public Ownership and the Solidarity City?” *Space and Polity* 24 (3): 314–316. 10.1080/13562576.2020.1787138.

[cit0033] Paul, F., and A. Cumbers. 2023. “The Return of the Local State? Failing Neoliberalism, Remunicipalisation, and the Role of the State in Advanced Capitalism.” *Environment & Planning A: Economy & Space* 55 (1): 165–183. 10.1177/0308518X211050407.

[cit0034] Paul, F., and A. Cumbers. Forthcoming. “Organizing for the Energy Transition: Labour, Energy Democracy and the Climate Emergency.” In *Handbook of Labour Geography*, edited by A. Herod. Cheltenham, UK: Edward Elgar.

[cit0035] Polanyi, K. 1957. *The Great Transformation* ((1944)). Boston: Beacon Press.

[cit0036] Public Futures. 2023. “Database”. Official website: https://publicfutures.org/en/cases

[cit0037] Rauprich, W. 2020. “30 Jahre Entsorgungsdienstleistungen im Ilm-Kreis: stabil, erfolgreich und jetzt kommunal.” *Technologie Region Ilmenau Arnstadt*, July 2. http://www.tria-online.eu/Newseintrag.17.0.html?&tx_news_pi1%5Baction%5D=detail&tx_news_pi1%5Bcontroller%5D=News&tx_news_pi1%5Bnews%5D=7255&cHash=cf93da87f2609bb90a18dff35d0bb5f7.

[cit0038] Rethmann, L. 2020. “ÖPP auf kommunaler Ebene: Infrastrukturprojekte und gemeinsame Unternehmen – Analyse von Fallbeispielen.” In *Öffentlich-Private Partnerschaften*, edited by M. Schäfer. Berlin: Springer. 259–312.

[cit0039] Rügemer, W. 2006. *Privatisierung in Deutschland: eine Bilanz*. Münster: Westphälisches Dampfboot.

[cit0040] Schäfer, M. 2020. “ÖPP – ein nationaler und internationaler Exkurs.” In *Öffentlich-Private Partnerschaften: Auslaufmodell oder eine Strategie für kommunale Daseinsvorsorge?*, edited by M. Schäfer and L. Rethmann. Wiesbaden: Springer Gabler. 49–74.

[cit0041] Scheler-Stöhr, D. 2017. “ÖPNV-Debatte wird zur Schlammschlacht”. *In* *Südthüringen*, November 3. https://www.insuedthueringen.de/inhalt.arnstadt-oepnv-debatte-wird-zur-schlammschlacht.56aba194-c92a-42c6-a789-53bf23c34524.html.

[cit0042] Schigold, A. 2017. “Kommunalisierung der Abfallwirtschaft im Ilm-Kreis.” In *Kommunale Abfallpolitik in Thüringen*, edited by C. Meyer. Erfurt: DAKT e.V. 10–12.

[cit0043] Technologie Region News. 2012. “Der Kreistag hat einene klaren Auftrag erteilt…”, August 14: http://www.tria-online.eu/Newseintrag.17.0.html?&tx_news_pi1%5Baction%5D=detail&tx_news_pi1%5Bcontroller%5D=News&tx_news_pi1%5Bnews%5D=2867&cHash=456f948b97a1c9fa292fd164fe823583.

[cit0044] Technologie Region News. 2015. “Kommunalisierung der Abfallwirtschaft im Ilm-Kreis ist perfekt.”, January 29: http://www.tria-online.eu/Newseintrag.17.0.html?&tx_news_pi1%5Bnews%5D=3584&tx_news_pi1%5Bcontroller%5D=News&tx_news_pi1%5Baction%5D=detail&cHash=fa24b8f4bdaf2cfc76df86a7d4d098a4.

[cit0045] UCLG (United Cities and Local Governments). 2022. *GOLD VI: Pathways to Urban and Territorial Equality*. Barcelona: UCLG.

[cit0046] Voigtmann, S. 2022. “Sie fällt auf: Petra Enders ist seit zehn Jahren Landrätin im Ilm-Kreis.” *MDR Thüringen*, 1 July: https://www.mdr.de/nachrichten/thueringen/sued-thueringen/ilmenau-ilmkreis/petra-enders-landraetin-jubilaeum-100.html.

[cit0047] Wagner, O., and K. Berlo. 2017. “Remunicipalisation and Foundation of Municipal Utilities in the German Energy Sector: Details About Newly Established Enterprises.” *Journal of Sustainable Development of Energy, Water and Environment Systems* 5 (3): 396–407. 10.13044/j.sdewes.d5.0152.

[cit0048] Warner, M. 2023. “Pragmatic Municipalism: Privatization and Remunicipalisation in the US.” *Local Government Studies* 1–19. Online first. 10.1080/03003930.2022.2162884.

[cit0049] Warner, M., and A. M. Aldag. 2021. “Re-Municipalization in the US: A Pragmatic Response to Contracting.” *Journal of Economic Policy Reform* 24 (3): 319–332. 10.1080/17487870.2019.1646133.

[cit0050] Weghmann, V. 2017. *Waste Management in Europe. Good Jobs in the Circular Economy?*. Brussels: European Public Service Union.

[cit0051] Weghmann, V. 2021. *Daseinsvorsorge und Rekommunalisierung*. Berlin: Rosa Luxemburg Foundation.

[cit0052] Wright, E. O. 2019. *How to Be an Anti-Capitalist in the 21^st^*. *Century*. London: Verso.

